# Notification and Isolation

**Published:** 1900-06-16

**Authors:** 


### NOTIFICATION AND ISOLATION.

In the midst of all the building of isolation hospitals which is going on at such a pace in various parts of the country, it is perhaps as well to be reminded that isolation is but one among the many influences which have resulted in lowering the zymotic mortality in this country. A careful letter, signed "Medicus," appears in a recent issue of the *Nottingham Guardian*, in which attention is drawn to the fact that the diminished loss of life from scarlet fever which has occurred in recent years has been general throughout the country, has by no means been confined to those parts in which isolation has been practised, and has not corresponded in time with the introduction of the practice of isolation. Moreover, he shows that the lowering of the mortality is by no means confined to scarlet fever, the disease in the management of which isolation has been mostly employed. The Notification Act was passed in 1889. Taking, then, the Registrar-General's returns from 1856 to 1890 in quinquennial periods, he shows that there was a steady and progressive lowering of the death-rate from scarlet fever in periods with which Notification Acts and isolation hospitals could have but little to do. The following are the figures which he gives:—

| | | |
|---|---|---|
| 1856 ... | ... 60 ... | ... 0·89 |
| 1861 ... | ... 65 ... | ... 0·98 |
| 1866 ... | ... 70 ... | ... 0·95 |
| 1871 ... | ... 75 ... | ... 0·76 |
| 1876 ... | ... 80 ... | ... 0·68 |
| 1881 ... | ... 85 ... | ... 0·43 |
| 1886 ... | ... 90 ... | ... 0·24 |

In the same period, he says, there was a similar diminution in the death-rate from diphtheria which was not maintained after the passing of the Notification Act. He also gives figures for the same groups of years, showing that in the mortality rate for phthisis and other tuberculous diseases, in which, of course, no sort of isolation had been practised, a similar diminution was exhibited, and still further he points out that grouping together all the seven principal zymotic diseases the same result is met with, and he urges that the great improvement which is sometimes claimed as the result of notification and isolation, must be due to other and much more widely-spread causes—causes which had begun to be operative before notification and isolation had come into force. Perhaps "Medicus" is wrong in believing that leading sanitarians do attribute the improvement which has taken place so exclusively as he suggests to isolation and notification, but in any case the figures which he gives are of much interest. Some three years ago the whole question was worked out very thoroughly by Dr. John T. Wilson,[1] who by very elaborate statistics, collected from many sources, showed the complete want of parallelism between the progress of isolation and the improvement of the scarlet fever mortality, and we do not think that his figures have yet been answered. Nevertheless, answered or not, we would not readily admit that notification and isolation, when carried out with reasonable common sense, are without influence for good. Looking at the question all round, we can hardly avoid the conclusion that some new influence for evil, from a sanitary point of view, has been introduced during recent years—perhaps universal and compulsory education—and that in consequence of this change in the conditions, the employment of isolation as an addition to other sanitary measures has failed to produce that marked effect upon statistics which would have otherwise resulted, a conclusion in which we are supported by the fact that measles, which still remains outside the Act in nearly all towns, shows an increasing instead of a diminishing mortality.

[1] Public Health, Feb., 1897.

